# Virtual reality as a tool to promote healthcare providers wellbeing in pediatric palliative care

**DOI:** 10.1186/s12913-025-13253-z

**Published:** 2025-10-03

**Authors:** Anna Marinetto, Valentina De Tommasi, Mariangela Rosa, Anna Santini, Caterina Carraro, Isabella Rosato, Pierina Lazzarin, Franca Benini, Anna Zanin

**Affiliations:** 1 Department of Women’s and Children’s Health, University of Padua, Padua, Italy; 2 University of Padua, Padua, Italy; 3 Unit of Biostatistics, Epidemiology and Public Health, DCTVPH, University of Padova, Padua, Italy

**Keywords:** Virtual reality, Pediatric palliative care, Anxiety, Stress, Healthcare workers, Risk of burnout

## Abstract

**Background:**

The pediatric palliative care (PPC) team’s mission is to assist and promote the highest quality of life for children with life-threatening and life-limiting illnesses and their families. The whole care of these patients implies sometimes challenging clinical and emotional situations. PPC providers may be exposed to psychological distress.

**Aims:**

The study’s objectives were to evaluate the psychological well-being of healthcare providers working in the Regional Center for Palliative Care and Pediatric Pain Therapy, including their stress levels, levels of depression, anxiety, and risk of burnout, and to determine whether practicing mindfulness through virtual reality can improve these items.

**Methods:**

The mindfulness intervention was delivered twice a week for four weeks in this prospective, non-randomized clinical study. Each participant received a 10-minute mindfulness-related session in 3-D virtual reality, for a total of eight exposures. Measures of emotional depression, anxiety, stress, and risk of burnout were assessed using the DASS-21 and Mini-Z questionnaires. Participants’ respiratory and heart rate were also monitored throughout each session. The treatment was evaluated using the DASS-21 at each timepoint, the Mini-Z at T0 and T3, and vital parameters at T1, T2, and T3 (T0 before the treatment, T1 at the end of the first week, T2 at the end of the second week, and T3 after completion of the last week’s treatment).

**Results:**

Pediatricians, nurses, allied healthcare professionals, and pediatric residents made up the 27 PPC healthcare practitioners enrolled. The median age was 47 years (IQR 36–50), and 85% of the participants were female. At the time of recruitment (T0), around 25% of individuals (*n* = 7; 25.93%) acknowledged a risk of burnout. Between T0 and T3, there was a significant shift in the DASS-21 scores for depression, anxiety, and stress (*p* <.05), indicating an improvement in the overall scores. The study of vital signs revealed that over the weeks, the heart and breathing rates had significantly decreased. It has also been demonstrated that the candidate’s mood significantly improved at T3 compared to the study’s beginning.

**Relevance to clinical practice:**

According to the current research, using virtual reality in a PPC team is a potential technology that may be helpful in lowering stress levels and the risk of burnout, resulting in significant improvements in the well-being of the healthcare personnel.

## Introduction

The field of Pediatric palliative care (PPC) requires multi-specialistic healthcare professionals able to improve and offer the best quality of life for children with life-threatening and life-limiting illnesses and their families [1].

These patients need support and long-term care, being provided adequate assistance for clinical, psychosocial and spiritual needs. In particular Pediatric Palliative care Healthcare delivery mode offers a global response to family and children’s need, where the main aim of care is no longer the full recovery, but offering the best possible quality of life [2].

The planning and implementation of each intervention must balance any risk and benefit complying with the child’s and family’s quality of life. Ethical reflection on the choice of treatment must be imperative for all healthcare professionals [3]. PPC providers may be exposed to complex and intense care experiences. The complexity and intensity of such experiences are linked by several factors including the establishment of strong, even long-standing relationships between the care team and the child/family, who may experience moments of severe distress and the need to deal with ethically complex issues involving the subject of a child’s life and death [4].

Frequent exposure to the death and suffering of patients being cared for can generate profound physical, cognitive, emotional, behavioral and spiritual repercussions in the health care worker.

In fact, as reported in literature, an increased risk among healthcare workers may develop issues such as anxiety, stress, compassion fatigue (CF) and burnout [5–8].

The emotional and psychological response that arises from such care experiences represents an area very often overlooked by the team itself [9]. When staff experience psychological stress and compassion fatigue, without adequate analysis of their experience, the risk of occupational burnout increases and quality of care decreases. Creating adequate support for healthcare staff is of upmost importance for both the patient and the pediatric palliative care team [10].

Within the context of the wellbeing of health-care professionals, recent studies have shown how virtual reality is a highly specialized and effective tool in the prevention and treatment of symptoms such as anxiety and stress [11, 12]. There has been a growing interest in the use of virtual reality for the treatment of stress and burnout for healthcare professionals since the covid 19 pandemic.

From a technological point of view, Virtual reality (VR) is an exclusively digital environment created by one or more computers that simulates actual reality and recreates it in a nontangible way. The created environment is conveyed to the senses through consoles that allow real-time interaction with everything produced within that world. In virtual reality, individuals interact with the virtual world using their bodies; this allows the perception of being in one place, even though physically located in another place [13, 14].

According to Kabat-Zinn, mindfulness is the ability to pay attention to the current moment in a non–judgemental attentive awareness [15].

Although the positive effects of Mindfulness in significantly reducing burnout, stress, and anxiety with improved clinical care have been widely demonstrated in the literature [6, 15], few data are available describing mindfulness-based intervention in the pediatric palliative care setting [16].

This study aimed to evaluate the psychological well-being of healthcare providers working in the Regional Center for Palliative Care and Pediatric Pain Therapy of Veneto (Padua, Italy) including their stress, depression, anxiety levels, and risk of burnout to determine whether practicing mindfulness through virtual reality can improve outcomes.

## Methods

### Setting

The project is a non-randomized clinical study that was implemented at the Regional Center for Palliative Care and Pediatric Pain Therapy, Pediatric hospice, located in the Department of Women and Children Health - University of Padua.

Data were collected between September and October 2023.

### Participants

Inclusion criteria for the study were as follows: being part of the pediatric palliative care team operating at the Padua Pediatric Hospice, not having neurological diseases (e.g., seizures), and not being pregnant at the time of signing the consent. The team is composed of doctors, nurses, and psychologists who work in direct contact with patients, providing care and assistance. Individuals were excluded from the study if they could not guarantee attendance at work for 4 consecutive weeks (e.g., due to already scheduled annual leave).

Informed consent was obtained from all study participants prior to their inclusion in the study. The participants were provided with detailed information about the purpose, procedures, and objectives of the study, as well as the potential risks and benefits. This information was delivered both verbally during the ward meeting and through a written consent form. Participants had the opportunity to ask questions and clarify any concerns before signing the consent form. The process ensured that all participants fully understood the study and voluntarily agreed to participate.

### Protocol

**Fig. 1:**
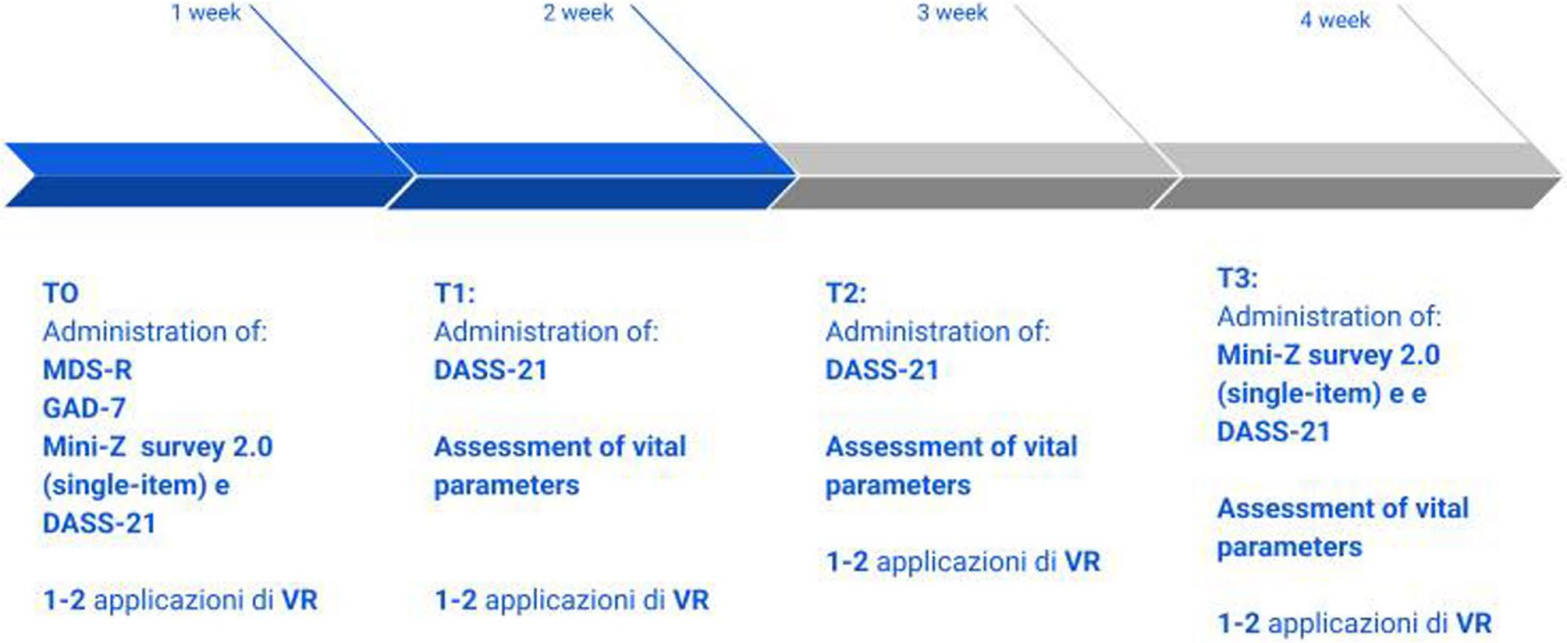
Timeline of the study

An overview of the intervention procedures and timeline is provided in Fig. 1.

Healthcare workers had to attend from one to two mindfulness-related sessions of 10 min in 3D virtual reality per week during their work shift. Data collection lasted four weeks.

Subjects were allocated in a specific room away from the clinical activity.

In the first session, defined time zero (T0), all participants completed three questionnaires: a demographic questionnaire, a Mini-Z survey 2.0 (the single item burnout question), the Depression Anxiety Stress Scales 21 (DASS-21); then they performed at least one VR session (with the option of repeating a second session three or four days after the first).

At time T1 (second week) and time T2 (third week) before the VR session, DASS-21 was filled in. During the last week of treatment (fourth week) at the end of the VR session (T3), both Mini-Z survey 2.0-the single item burnout question and the DASS-21 were repeated prior to the last two virtual reality sessions (see Fig. 1).

Meanwhile, heart rate, saturation and respiratory rate were also monitored during the treatment at minutes 1–5 and 9 of the VR session.

### Measures/Instruments

#### Questionnaires

**Demographic characteristics** At the beginning of the study, participants were asked to fill in a demographic questionnaire. Individuals indicated their gender, age, profession, professional seniority, work in shifts, serious events in the last four weeks, and whether they lived alone.

**Working well-being** The assessment of working well-being within the pediatric palliative care team was conducted using the Mini-Z Survey 2.0 (single-item). The most recent version of the Mini-Z survey is a validated, brief, and effective tool to measure the “temperature” of the work environment. It was developed for use in healthcare professionals (physicians and advanced practice providers) and has been adapted for use in nurses, residents, medical students, executive leaders, and teams both inside and outside of healthcare. Many authors have used and validated this tool in populations of student/resident/internist physicians [17, 18], general practitioners [19], primary care staff, and clinical associates, such as LPNs and medical technicians [20]. Although no studies have applied this instrument to healthcare professionals in adult and/or pediatric palliative care, one research study has longitudinally examined the risk of burnout among frontline physicians in a New York City hospital during the COVID-19 pandemic [21]. The Mini-Z was chosen to identify the burnout risk of workers on the pediatric palliative care team, rather than for promoting burnout measurement screening, for which the Maslach Burnout Inventory (MBI) would have been more appropriate. Furthermore, the Mini-Z was selected for its suitability in terms of application (no license charges), length, and its recommendation for surveys or research where administration time is limited.

Mini-Z Scoring and Classification Criteria: The Mini-Z survey (2.0) includes a single-item question aimed at assessing burnout risk on a 5-point Likert scale, where participants rate their level of burnout as follows:

1-“I am in total burnout”.

2-“I always have burnout symptoms; they are persistent”.

3-“I am at the beginning of burnout”.

4-“I feel under stress, but I don’t feel burnout symptoms”.

5-“I never felt burnout.”

For the purposes of this study, these responses were categorized as follows:

Medium-High Risk of Burnout: Participants selecting options 1, 2, or 3.

Low Risk of Burnout: Participants selecting options 4 or 5.

Burnout risk was assessed at two time points, T0 and T3, to evaluate changes over the course of the intervention. Statistical analyses were conducted to compare the distribution of burnout risk levels between these time points, using frequencies, percentages, and paired tests where appropriate. Additionally, changes in burnout severity (e.g., transitioning from “In total burnout” to “Beginning of burnout”) were analyzed descriptively to explore trends within the sample.

**Anxiety**,** depression**,** and stress** The Depression Anxiety Stress Scales 21 (DASS-21) was used to measure anxiety, depression, and stress. This psychometric self-assessment test, derived from the original Depression Anxiety Stress Scales [22], is composed of 21 items measured on a 4-point Likert scale (from “0 - Not at all applies to me” to “3 - It applies a lot or most of the time to me”). The DASS-21 has demonstrated good psychometric properties and reliability, with a Cronbach’s alpha of 0.80, and its Italian version has shown strong reliability in both clinical and non-clinical populations [23]. Before each treatment, participants reported their mood on a scale from zero to ten, where ten represented a complete state of well-being. This self-reported mood scale was integrated into the mindfulness-related session.

**Physiological parameters** Measurements of oxygen saturation, heart rate (HR), and respiratory rate (RR) were conducted using a Masimo™ pulse oximeter. These parameters were recorded to monitor participants’ physiological responses during the intervention.

The detection of vital parameters was conducted during the application of virtual reality at three specific timepoints: T1, T2 and T3. Specifically, the measurements were taken at the beginning (minute 1), in the middle (minute 5) and at the end (minute 10) of each treatment session, resulting in a total of 9 observations for each participant throughout the study. These timepoints were chosen to capture potential physiological changes occurring at key moments during the mindfulness-based VR experience. The 1 st minute was chosen to establish a baseline measurement before the participant engaged fully in the mindfulness exercises. The 5th minute was identified as a critical point to evaluate the physiological impact of controlled breathing exercises, which were introduced at this stage of the session. The 10th minute provided data on the cumulative effects of the entire session on vital parameters. This framework allowed us to track dynamic changes in HR and RR over the course of each session and assess the efficacy of the intervention in promoting relaxation and mindfulness.

For timepoint T2, the mean and median values of HR and RR readings were calculated. Additionally, differences in HR and RR across the three measurements (I: 1 st minute; II: 5th minute; III: 10th minute) were compared, as shown in Table 3.

Before each treatment, the subject reported his/her own mood with a score from zero to ten, where ten represents a complete state of well-being. The mood item was integrated into the virtual reality program itself. Participants were presented with a set of facial expressions (emojis or faces) within the VR interface, and they were asked to select the face that best represented their current state of well-being at that precise moment. This visual and interactive approach was designed to simplify and enhance the self-assessment process for participants during the mindfulness-related sessions.

#### Virtual reality device and experience

Oculus™ is a virtual reality (“VR”) device (by Meta) that offers users a 360-degree immersive view of a three-dimensional virtual reality world.The system includes a Visor, two Oculus Sensors and a pair of Oculus Touch controllers. The Visor and sensors are connected to a workstation (PC) via USB cables. For added user safety, Oculus was also connected to a tablet that allowed the content seen by the user to be viewed via mirroring by staff monitoring.

The software TRIPP (© TRIPP, INC.) Free Demo allowed each participant to be exposed, for about 10 min, in a relaxing and immersive environment with controlled breathing exercises, surrounded by slow and calm music that helps to induce relaxation.

### Statistical analysis

In the descriptive analysis, the sample characteristics were presented using mean (standard deviation) and median for continuous variables and absolute and relative frequencies for categorical ones.

We used either parametric and nonparametric tests (after checking for normality of the distributions) for paired samples to assess the presence of significant differences among all the time-points available. When only 2 time-points were available, we used the Wilcoxon test or the Student’s t-test for paired samples. When considering more than 2 time-points, we used the repeated measures ANOVA test together with the Dublin-Conover test for pairwise comparisons. A p-value < 0.05 was considered statistically significant. The analyses were conducted using R software (R statistical analysis, https://www.R-project.org/).

## Results

### Sample characteristics

A total of 27 enrolled individuals were recruited from healthcare workers in Pediatric palliative Care and Pain Service of Padua. The sample consists of 27 participants (*n* = 23 female, 85%) with a median age of 47 (IQR 36–50) years. In detail, the sample is represented by *n* = 16 nurses (59%), *n* = 4 physicians (15%), *n* = 3 psychologists (11%), and *n* = 4 health and social workers and administrative clerks (15%). Some participants, *n* = 15 (56%) do not work shifts but only daytime.

*N* = 23 participants (85%) reported that they do not live alone. Lastly, participants were asked if they had experienced a ‘serious event’ (*n* = 6; 22%) which affected their lives in the previous four weeks (Table 1).

**Table 1:** Participant Demographics

	*N* = 27 *N* (%)
Gender	
M	4 (15%)
F	23 (85%)
**Profession**	
Nurse	16 (59%)
Psychologist	3 (11%)
Administer clerks/health workers/	4 (15%)
Physician	4 (15%)
**Living alone**	
No	23 (85%)
Yes	4 (15%)
**Work in shifts**	
No	15 (56%)
Yes	12 (44%)
**Serious events in the last four weeks**	
No	21 (78%)
Yes	6 (22%)
**Age (median**,** IQR)**	47 (36, 50)

### Mini-Z questionnaire

**Fig. 2:**
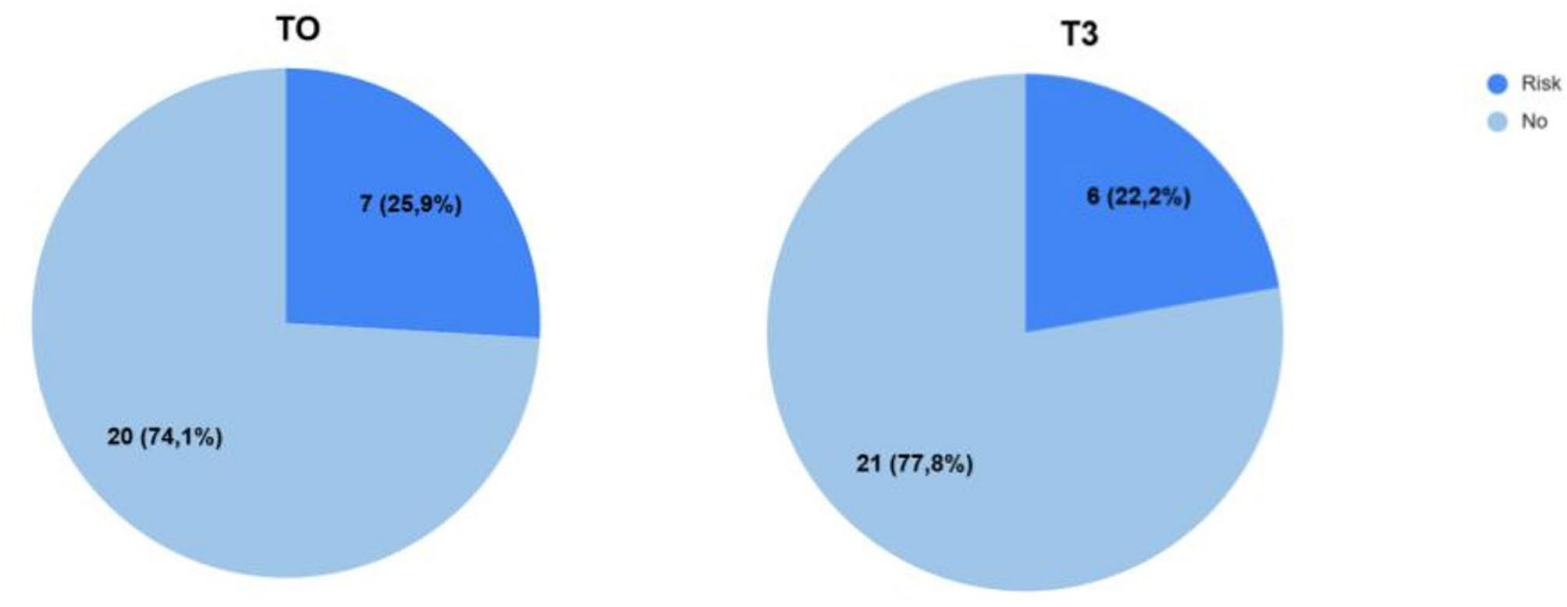
2a (T0) E 2b (T3) Mini-Z scorings

As described in the Methods section, the Mini-Z survey was used to assess burnout risk among healthcare workers. Frequencies and percentages of burnout risk were analyzed based on the criteria outlined in Nagasaki et al. (2022) [17]. At T0, seven subjects (25.9%) exhibited medium-high burnout risk, while at T3, this number decreased to six subjects (22.2%) (see Fig. 2). Continuous scores obtained for the Mini-Z questionnaire at T0 and T3 were compared using the Wilcoxon rank test, which revealed no statistically significant difference (*p* =.863) between the two time points. Specifically, a total of 20 participants (74.1%) obtained the same score at both time points, while the small differences in scores among the remaining participants were not statistically significant.

Analysing by severity, of the 7 participants at time T0, 1 operator (3.7%) self-rated “in total burnout “, 5 participants (18.5%) self-assess themselves “Beginning of burnout,” 1 participant (3.7%) self-rated themselves persistently symptomatic of burnout “I always have symptoms of burnout.” While looking at the data at T3 time, it can be seen how only 1 participant (3.7%) self-assesses himself/herself to be self-rated “In total burnout” while the remaining 5 participants (18.5%) as “Initiated burnout.”

These findings suggest a reduction in burnout risk when comparing the number and severity of cases between T0 and T3.

### DASS-21 questionnaire

**Fig. 3:**
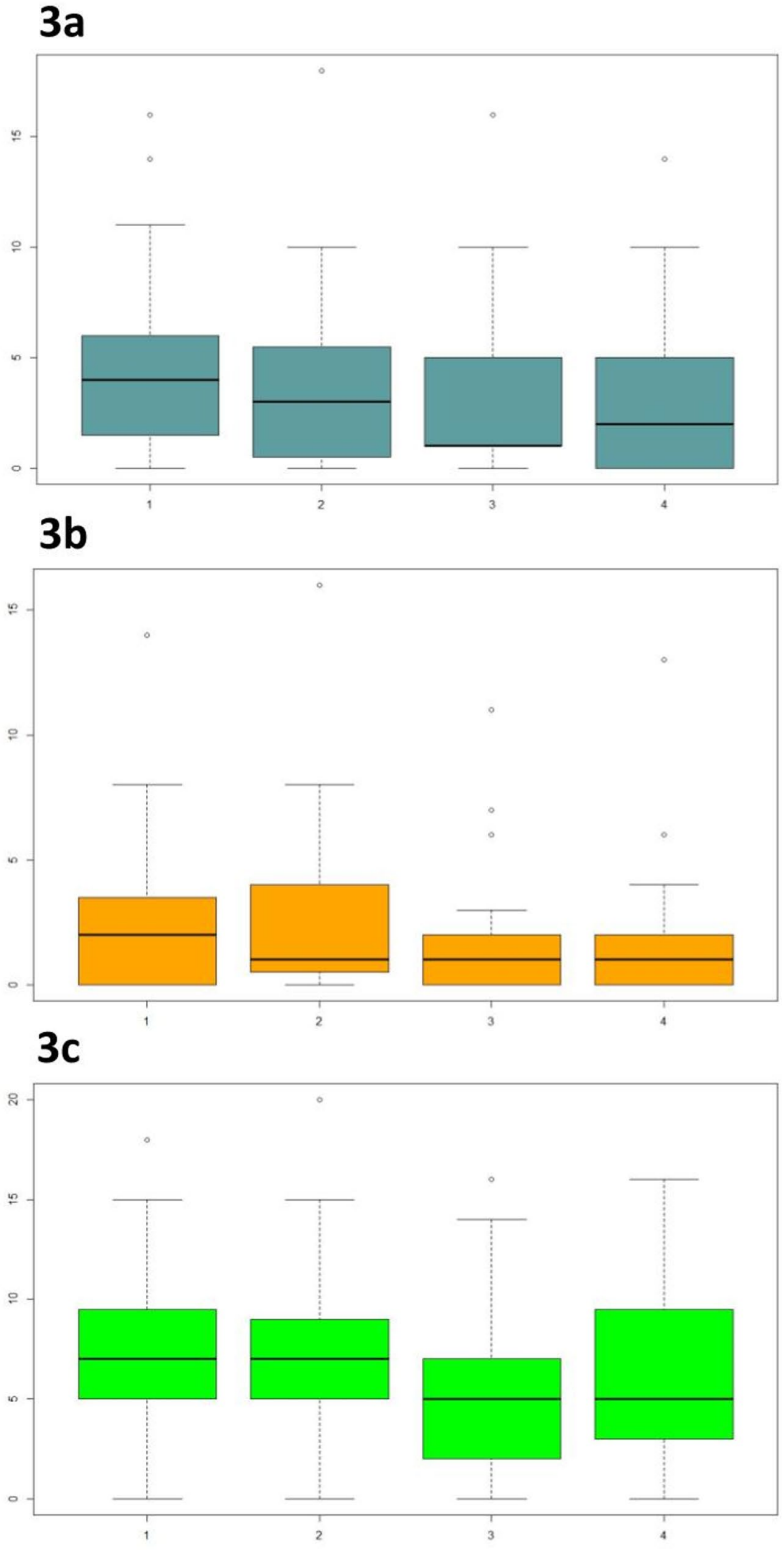
Plot box: DASS-21 questionnaire mean values at T0, T1, T2 and T3 Legenda. 3a, DASS-21 depression; 3b, DASS-21 anxiety; 3c, DASS-21 stress

The DASS-21 scoring system detailed in the Methods section was used to evaluate psychological well-being. It was administered at the beginning of T0, T1, T2 and T3 (Fig. 3). Mean values were calculated with respect to the items “depression” “anxiety” and “stress” at all 4 time-points considered.

The comparison between the scores obtained at DASS-21 at T0 and T3 highlights a significant difference in depression (T = 1.78; *p* <.001), anxiety (T = 1.04; *p* =.010), stress (T = 1.56; *p* =.050) and the “total score” (T = 4.37; *p* =.002) see Table 2.

**Table 2:** Scores DASS-21 T0-T3

	Mean (T0)	Mean (T3)	Average difference	*p*-value
DASS-21 depression	4.63	2.85	1.78	< 0.001
DASS-21 anxiety	2.78	1.74	1.04	0.010
**DASS-21 stress**	**7.93**	**6.37**	**1.56**	**0.050**
**DASS-21 total**	**15.33**	**10.96**	**4.37**	**0.002**

These findings demonstrate the effectiveness of mindfulness-based intervention.

### Detection of vital parameters

As outlined in the Methods, the efficacy of virtual reality was also evaluated through the analysis of vital parameters in terms of reduction of heart rate and respiratory rate as an indicator of the effectiveness of mindfulness [24, 25].

**Table 3:** Pairwise Comparisons (Durbin-Conover)

Heart rate	HR T2 I	HR T2 II	HR T2 III
>Mean (SD) Median (IQR)	71.7 (10.4) 70 (15.5)	70.1 (8.15) 70 (11.0)	68.7 (9.10) 68 (12.0)
**Pairwise Comparisons (Durbin-Conover)**		**p-value**	
HR T2 I	HR T2 II	0.217	
HR T2 I	HR T2III	< 0.001*	
HR T2II	HR T2 III	0.019 *	

**Respiratory Rate**	**RR T2 I**	**RR T2 II**	**RR T2 III**
Mean (SD) Median (IQR)>	15.0 (3.69) 14 (4.00)	17.1 (3.31) 17 (4.00)	15.9 (3.51) 16 (5.50)
**Pairwise Comparisons (Durbin-Conover)**		**p-value**	
RR T2 I	RR T2 II	< 0.001 *	
RR T2 I	RR T2III	0.312	
RR T2 II	RR T2 III	0.010 *	

However, regarding the parameter “Saturation,” the comparison of the averages did not yield statistically significant results (*p* =.650). Similarly, for the T3 survey, the mean and median were calculated, and the values for heart rate and respiratory rate were compared across the three available measurements (I: 1 min; II: 5 min; III: 10 min), as shown in Tables 3 and 4. The comparison of the three surveys (T1, T2, T3) revealed a progressive reduction in both heart rate and respiratory rate, particularly at the measurement of T2 (5th minute), during which participants were guided through a controlled breathing exercise.

**Table 4:** Pairwise Comparisons (Durbin-Conover)

Heart Rate	HR T3 I	HR T3 II	HR T3 III
Mean (SD) Median (IQR)	71.3 (8.36) 70 (11.5)	68.8 (9.87) 68 (11.0)	70.3 (10.9) 69 (12.5)
**Pairwise Comparisons (Durbin-Conover)**		**p-value**	
HR T3 I	HR T3 II	0.008 *	
HR T3 I	HR T3 III	0.046*	
HR T3II	HR T3 III	0.469	

**Respiratory Rate**	**RR T3 I**	**RR T3 II**	**RR T3 III**
Mean (SD) Median (IQR)	14.3 (3.34) 15 (4.50)	16.4 (2.98) 16 (4.00)	15.6 (3.36) 15 (4.50)
**Pairwise Comparisons (Durbin-Conover)**		**p-value**	
RR T3 I	RR T3 II	0.038 *	
RR T3 I	RR T3 III	0770	
RR T3II	RR T3 III	0.071 *	

For RR (Table 5): A significant difference was observed, with a reduction noted between the baseline measurement at the start of the session and the 5th minute, as well as between the 5th minute and the final baseline measurement at the end of the session.

**Table 5:** Pairwise Comparisons (Durbin-Conover)

Pairwise Comparisons		*p*-value T1	*p*-value T2	*p*-value T3
*Heart Rate*				
HR I	HR II	0.209	0.217	0.008 *
HR I	HR III	0.833	< 0.001*	0.046*
HR II	HR III	0.294	0.019 *	0.469
*Respiratory Rate*				
RR I	RR II	0.020 *	< 0.001 *	0.038 *
RR I	RR III	0.941	0.312	0770
RR II	RR III	0.024 *	0.010 *	0.071 *

For HR (Table 5): At T1, no significant differences were detected. However, differences became apparent at later time points, suggesting that the mindfulness exercises were performed effectively. This was further evidenced by a progressive improvement in the participants’ ability to control their breathing.

### Mood (state of mind at the time of the reality session virtual reality)

Mood monitoring was assessed twice, before and after the mindfulness session, in each of the 4 weeks of the study. A statistically significant difference was underlined between T0 and T3 mood as reported in Table 6.

**Table 6:** Mood T0-T1-T2-T3

Mood T0			
First VR session	Mean (DS)	Median	*p*-value
Mood1	6.35 (1.35)	6.0	< 0.001 (Wilcoxon)
Mood2	8.00 (1.27)	8.0	

**Second VR session**	**Mean (DS)**	**Median**	**p-value**
Mood1	6.35 (1.46)	6.0	< 0.001 (t Student)
Mood2	8.13 (1.06)	8.0	

**Mood T1**			
**First VR session**	**Mean (DS)**	**Median**	**p-value**
Mood1	6.41 (1.42)	7.0	< 0.001 (Wilcoxon)
Mood2	7.89 (1.01)	8.0	

**Second VR session**	**Mean (DS)**	**Median**	**p-value**
Mood1	6.78 (1.24)	7.0	< 0.001 (t Student)
Mood2	8.13 (1.06)	8.0	

**Mood 2**			
**First VR session**	**Mean (DS)**	**Median**	**p-value**
Mood1	7.11 (1.15)	7.0	< 0.001 (t Student)
Mood2	8.56 (0.85)	8.0	

**Second VR session**	**Mean (DS)**	**Median**	**p-value**
Mood1	6.59 (1.42)	7.0	< 0.001 (t Student)
Mood2	8.29 (0.98)	8.0	

**Mood 3**			
**First VR session**	**Mean (DS)**	**Median**	**p-value**
Mood1	6.30 (1.38)	6.0	< 0.001 (T Student)
Mood2	7.70 (1.10)	8.0	

**Second VR session**	**Mean (DS)**	**Median**	**p-value**
Mood1	7.10 (1.20)	7.0	< 0.001 (T Student)
Mood2	8.90 (0.57)	9.0	

## Discussion

This is the first study applying virtual reality as a mindfulness-based intervention modality for health care professionals in the pediatric palliative care setting.

The majority of studies regarding the use of virtual reality for the treatment of stress and burnout among health care professionals date to covid and post-COVID19 period.

Beverly et al. in 2022 conducted a pilot study in three United States units of COVID-19 [26]. The results suggest that the application of a three-minute simulation of a nature scene through VR, reduces the subjective stress of healthcare workers on the frontline workers in the short term. Moreover, the intervention appears to be accessible to health care workers even during their work shift. The impact in the long term will be an area of interest for further research.

In a randomized controlled trial conducted by Pallavicini et al. (2022) proposed to investigate the efficacy and acceptability of brief home-based virtual reality training for the management of stress and of anxiety during the COVID-19 crisis in a sample of practitioners Italian healthcare workers (this study is still unfinished) [27].

In addition, Nijland et al. (2021) reported that virtual reality represents an effective tool to reduce the perceived stress immediately among the staff of an intensive care unit in the Netherlands. During working shifts, nurses were allowed to use VR as a 10-minute break during their shift, inducing a positive emotional state [28].

In our setting we decided to use a VR mindfulness-based intervention as a support for the healthcare professionals as a tool regularly integrated in their job during their shifts. The program was safe and easy to apply, and, although the treatment took place during the employee’s working hours, it did not affect the clinical activities.

In addition, there were no cases of drop-outs during the course of the project. No participant reported any undesirable effects, such as nausea or dizziness; we hypothesize that the administration of relaxing content and exercises of breathing minimized the potential negative effects of the virtual experience.

The primary aim of this study is to assess the psychological well-being at work of the sample surveyed and to see whether the tool of virtual reality decreases the risk of burnout, but also stress, anxiety and depression.

Considering the risk of burnout of the operators, *n* = 7 of participants (26%) presented a risk of burnout during time T0. At the end of trial, the score dropped to *n* = 6 (22%). Therefore, despite there being a trend in lowering the risk of burnout with the treatment of virtual reality, further studies with bigger samples are needed.

Despite the lack of literature, we found that the application of the Mini-Z (single-item) allowed us to take a “real time” and quick snapshot through a self-assessment of the perceived risk of burnout by each individual health worker on the PPC team. In addition, the application of Mini-Z allowed us to find how the use of a short survey reduced the load and timing for participants in filling out the instrument tools by giving more space to the experiential part.

We also investigated if there was an association of burnout and participant’s specific serious events in personal life.This highlights participants’ self-assessments of burnout are independent and not influenced over time by stressors outside of work.

Relatively to the psychological well-being in terms of anxiety, depression and stress investigation, the data show how, during the 4-week experimental timeframe, there was an improvement in the psychological variables investigated at all time points considered. These results are consistent with studies showing that virtual reality with the use of mindfulness programs are effective in reducing states of anxiety and stress [6, 29]. In particular mindfulness based interventions are shown as effective in reducing these particular psychological disorders (Ghawadra/Guillaumie). The decrease of respiratory rate and heart rate which increase as the user becomes familiar with mindfulness probably contribute to a reduction of stress and promotes a state of calm. It is hypothesized that this change, which occurred at the end of the experience, resulted from learning the technique of breathing as shown by [29].

A particular attention to these important aspects related to behaviors of healthcare professionals, received media attention and have been studied in the last few years during and right after the emergency phase of the COVID19 pandemic [30]. Despite specific considerations related to the pandemic, physicians and allied healthcare professionals often have high levels of stress, anxiety and depression [30–32] due to the intrinsic demanding nature of their work. Many studies report how healthcare workers suffer from work-related or occupational stress often resulting from high expectations coupled with insufficient time, skills and/or social support at work, and this is particularly frequent in pediatric high dependency units as our PPC unit [33–35] or pediatric and neonatal intensive care units [36]. Psychological disorders should be recognized since they may compromise workers’ mental health, negatively impacting their quality of life but also service systems. Because the costs of stress and burnout are high due to increased absenteeism and turnover, it is crucial that hospital policymakers and managers take action by promoting measures such as surveillance, monitoring, and psychological support to increase the resilience of healthcare workers, limiting stress and anxiety, and allowing them to maintain their performance at work.

In recent years, the interest in the quality of life of the healthcare professional is increasing, becoming the care of the practitioner a real ethical and deontological duty [37].

The emotional impact and the stressful work environment that PPC workers experienced are often considered responsible for situations of severe burnout [9]. They are a potentially high risk population due the difficulties related to death and incurable disease and they deserve supervision, sharing, and support.

Although the number of participants was small, the results of the study showed that the use of virtual reality, within the team of the UOC Veneto Regional Center for Pediatric Pediatric Palliative Care and Pain Therapy, is a valuable tool in ensuring an improvement in the well-being of the group. The small size of the cohort does not make the results sufficiently generalizable, and further studies with larger cohorts are needed. A second important limitation was the absence of studies that applied the chosen tools on health care workers in adult and pediatric palliative care.

Despite the fact that the virtual content was administered for just 10 min per session, this mindfulness-based intervention proved helpful in reducing anxiety, depression and stress. Such a short time frame repeated regularly, produced a state of relaxation with instant beneficial consequences, resulting in a positive emotional state that is an integral aspect of the virtual experience. It could be interesting to include a follow-up that serves as the foundation for a potential longitudinal research intended to evaluate the long-term efficacy of virtual reality in lowering anxiety, stress, and depression.

## Conclusion

This project represents the first study to evaluate the effectiveness of virtual reality in terms of reducing stress, anxiety and depression in a team of pediatric palliative care professionals. The positive impact on daily activity highlighted by our results support that this kind of intervention could be an effective, feasible and sustainable tool to support clinical work in high intensity settings.

Further studies are needed to optimize the strategies aimed at improving the well-being and enhancing satisfaction among PPC providers.

## Data Availability

Research data are available from the corresponding author upon reasonable request.

## Declarations

### Trial registry information

ClinicalTrials.gov Identifier NCT06247618. Registered February 07, 2024.
